# Magneto-Optical Indicator Films: Fabrication, Principles of Operation, Calibration, and Applications

**DOI:** 10.3390/s23084048

**Published:** 2023-04-17

**Authors:** Lev Dorosinskiy, Sibylle Sievers

**Affiliations:** 1 TUBITAK National Metrology Institute (TUBITAK UME), Dr. Zeki Acar Cad. No.1, Gebze 41470, Kocaeli, Turkey; 2 Physikalisch-Technische Bundesanstalt (PTB), Bundesallee 100, D-38116 Braunschweig, Germany; sibylle.sievers@ptb.de

**Keywords:** MOIF, Faraday effect, magneto-optics, magnetic sensors, local magnetic field measurements, quantitative, calibration

## Abstract

Magneto-optical indicator films (MOIFs) are a very useful tool for direct studies of the spatial distribution of magnetic fields and the magnetization processes in magnetic materials and industrial devices such as magnetic sensors, microelectronic components, micro-electromechanical systems (MEMS), and others. The ease of application and the possibility for direct quantitative measurements in combination with a straightforward calibration approach make them an indispensable tool for a wide spectrum of magnetic measurements. The basic sensor parameters of MOIFs, such as a high spatial resolution down to below 1 μm combined with a large spatial imaging range of up to several cm and a wide dynamic range from 10 μT to over 100 mT, also foster their application in various areas of scientific research and industry. The history of MOIF development totals approximately 30 years, and only recently have the underlying physics been completely described and detailed calibration approaches been developed. The present review first summarizes the history of MOIF development and applications and then presents the recent advances in MOIF measurement techniques, including the theoretical developments and traceable calibration methods. The latter make MOIFs a quantitative tool capable of measuring the complete vectorial value of a stray field. Furthermore, various scientific and industrial application areas of MOIFs are described in detail.

## 1. Introduction

Magneto-optical (MO) imaging techniques date back to the 19th century, when Oersted, Davy, and Ampere used iron particles to visualize magnetic fringe fields around a permanent magnet. In its modern form, known as Bitter magnetic decoration, this technique was first applied by Bitter, Hamos, and Thiessen in 1931. Further development of magneto-optical imaging was stimulated by studies of superconductors, when the Faraday effect [1] was first applied for this purpose using phosphate glasses and films of EuS, EuF_2_, and EuSe [2,3]. The application of phosphate glasses in 1957 became a strong breakthrough in the magneto-optical imaging as it allowed the visualization of magnetic field strength, not just the fringe patterns, for the first time. However, as the Verdet constant of such glasses is quite low, the magneto-optical contrast obtained was weak and a thick glass layer had to be used to increase it, which resulted in a low spatial resolution. EuS, EuF_2_, and EuSe films, on the contrary, have a large Verdet constant (especially EuSe films); thus, a thin film (below 1 μm) can produce sufficiently high MO contrast which allows one to achieve high spatial resolution near the optical resolution limit. However, such films have to be deposited directly on the studied sample, which makes the whole procedure difficult and time-consuming. Moreover, these films exhibit magneto-optical properties only below ~15 K, which substantially limits their area of application. Another very widely used technique is the magneto-optical Kerr effect (MOKE) [4,5,6,7,8,9]. This technique does not use any kind of magnetic coating, but the MO effect arises from the interaction of polarized light with the sample itself. Thus, MOKE can provide a very high spatial resolution up to the optical limit. The downsides are that the samples generally require special surface preparation, and the MO signal cannot be calibrated with respect to the magnetic field as there is no way to measure a reference signal in the absence of a sample. There are also more exotic approaches, such as using magnetotactic bacteria [10,11] and ferrofluid films [12]. Although successful in visualizing magnetic microstructures, these techniques cannot be calibrated; thus, they cannot be used for quantitative measurements and are not suitable for standardization [10,11,12].

Quantitative, sample-independent, and fast-calibrated measurements of local magnetic fields can be performed using rare-earth iron garnet magneto-optical indicator films (MOIFs) grown epitaxially on gallium gadolinium garnet (GGG) substrates. The application of these films is easy since they are self-supported and allow for the direct calibration of the local light intensity with respect to the magnetic field. This makes them suitable for selection as a standard technique which is both traceable and can be easily applied in various industrial processes. In this review, we shall discuss MOIFs in detail, including the history of their development and applications, preparation techniques, calibration approaches, and various areas of application. In previous years, several review papers have already been published on the MOIF technique, the most comprehensive one probably being ref. [13]. However, substantial theoretical advancements in understanding the physics of MOIFs have recently been made [14,15], which, in turn, have led to new developments in MOIF calibration and quantitative measurement techniques. As a result, new advanced industrial applications have been demonstrated. That is why now seems to be the right time for a comprehensive review that covers all recent advancements and, thus, presents a complete, exhaustive overview of the theory and applications of MOIFs.

In the following chapters, we will first describe the available types of MOIFs, the physics behind them, and their basic properties. Then, we will delve into the preparation technique and the process parameters that determine the type of MOIF produced. Then, we will describe in detail the existing calibration approaches and measurement techniques used for quantitative measurements of local magnetic fields. Finally, we will speak about the diverse applications of MOIF sensors, trying to cover all major areas of their scientific and industrial applications. The present review cites all the major landmarking works published on the basics of MOIFs and selected representative works on their typical applications.

## 2. Perpendicular and in-Plane MOIFs

Ferrite garnets {R_3_^3+^}[Fe_2_^3+^](Fe_3_^3+^)O_12_ have a cubic structure with rare-earth and iron ions occupying different positions ({R} dodecahedral, [Fe] octahedral, and [Fe] tetrahedral) between oxygen ions [13]. They are grown using liquid-phase epitaxy on GGG substrates. The properties of such films can be altered substantially through isomorphic substitutions of atoms in the three sublattices as well as by changing the substrate and the growth conditions (which varies the lattice mismatch between the film and substrate and thus changes the magnetoelastic anisotropy).

Ferrite garnets are isolating ferrimagnets with a net magnetization, ***M***. Their magnetization will arrange itself in such a way as to minimize the free energy density

(1) $$F=F_{m}+F_{d}+F_{A}=-\mu_{0}\left(M\cdot H\right)-\frac{\mu_{0}\left(M\cdot H_{d}\right)}{2}+K_{u}sin^{2}\theta +F_{c}$$

Here, *F*_m_ = −*μ*_0_ (***M∙H***) is the Zeeman energy of the magnetization in the external field ***H***, $F_{d}=-\mu_{0}\left(M\cdot H_{d}\right)/2$ is the energy in the self-demagnetizing field ***H_d_***, and *F*_A_ is the anisotropy energy (magnetocrystalline or induced) which consists of the uniaxial anisotropy $K_{u}sin^{2}\theta$ (*θ* is the angle between ***M*** and the anisotropy axis) and cubic anisotropy *F*_c_. In ferrite garnets, cubic anisotropy is very low and can be neglected [14].

As a result, the magnetic structure is determined by the so-called quality factor [13]

(2) $$Q=\frac{2K_{u}}{\mu_{0}M_{S}^{2}}\;$$

which is the ratio between the energy of magnetic anisotropy and maximum energy density due to shape anisotropy, $\mu_{0}M_{S}^{2}/2$. For *Q* > 1, the material will show uniaxial anisotropy with the easy axis’s direction being normal to the plane of the film, while for *Q* < 1, the magnetic moments will tend to be oriented in the plane of the film due to the effect of the demagnetizing field [13].

Perpendicularly magnetized films typically divide into magnetic domains to minimize the stray field energy. These domains reveal a labyrinth structure in zero external field. In increasing perpendicular fields, the domains with the opposite magnetization orientation shrink and form magnetic “bubbles”. That is why such films are also called “bubble films”. Back in the 1970s, they were intensively studied in an attempt to develop so-called magnetic bubble domain memories [16,17,18]. Such films can also be used for MO observations as the domains with the opposite magnetization orientation look differently if polarizers in the microscope are slightly uncrossed [19,20,21,22,23,24,25,26,27]. The interest towards them increased dramatically in the early 1990s after the discovery of high-temperature superconductors because they became a practical tool to study magnetization processes in HTSCs (see the references above). The obvious downside of bubble films is the spatial resolution being limited by the domain width. Another downside is their insensitivity to low fields below the coercive field H_C_, when the domain walls do not move due to pinning. At the same time, bubble indicators have the advantage that they can operate as optical contrast “amplifiers” and, thus, detect very weak contrast which may be impossible to observe using other tools, for example, in the case of domains in electrical steels [27]. In cases when the observed magnetic structure is large compared to the typical domain width in perpendicular MOIFs (which can vary from 5–10 μm down to the sub-micrometer scale), low magnification can be used to perform observations in an overview mode [27]. Then, individual domains in the MOIF are not visible and the MO contrast varies smoothly with magnetic field due an averaging over the varying ratio of up- and down-magnetized domains. In that case, the MOIF response can be calibrated with respect to magnetic field and, thus, quantitative local measurements can be performed [27].

If Q > 1, the magnetization will be confined within the film plane or may have a small out-of-plane component. In that case, there are either no domains in the film or very large, several-millimeters-wide domains occur, which normally do not interfere with the magnetic structure being observed. Magneto-optical contrast arises in this case due to the Faraday effect induced by the rotation of the magnetization vector out of the film plane under the effect of a perpendicular external field [28,29,30]. Such in-plane MOIFs have a number of advantages compared to perpendicular magnetized indicators: as there are no domains (or the domains are much larger than the observed magnetic structure), much higher spatial resolution can be achieved. The magnetization rotation process shows no coercivity, which makes such MOIFs sensitive to very low fields, without hysteretic effects. Moreover, as will be discussed below, there is an analogue optical scheme that makes the response of in-plane MOIFs linear with respect to the magnetic field. This makes the calibration procedure very simple and robust [15]. Due to these advantages, in-plane MOIFs are most suitable to be used as a standard technique and, thus, were chosen for this purpose by many research groups. In the following, we shall explore in more detail the various aspects of in-plane MOIFs, such as their fabrication, properties, calibration procedures, and applications.

## 3. In-Plane MOIFs’ General Properties

**Figure 1:**
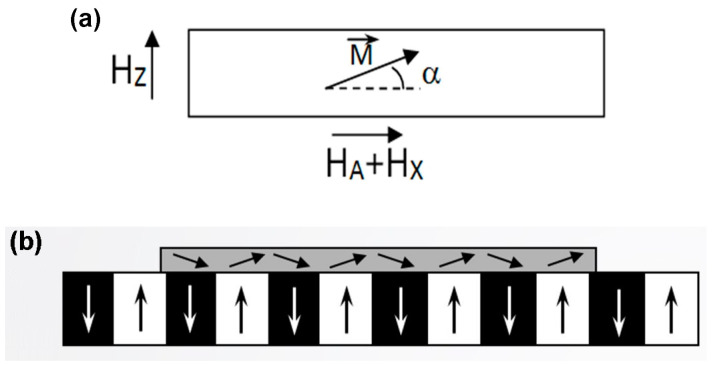
(**a**) Rotation of magnetization vector in MOIF under an external magnetic field. H_A_ is the anisotropy field of the MOIF; (**b**) Rotation of magnetization in MOIF under the effect of the sample’s stray field [15].

As the magnetization vector M in in-plane MOIFs is confined within the film plane when no external field is applied (or may also be slightly tilted), the MO response arises due to the Faraday effect produced by the normal component of M  when it turns toward the normal direction under the action of an external magnetic field [28,29,30], as illustrated in Figure 1.

The normal component of ***M*** can, thus, be detected optically in light transmitted through the MOIF via changes in the light polarization induced by the Faraday effect. A magneto-optical contrast can, thus, be generated in crossed polarizers, for example in a polarizing microscope [28,29,30] or using a low-magnification overview system for larger samples [31]. As the magnetic samples observed using MOIFs are generally nontransparent, a microscope or an optical system working in reflection mode should be used and, thus, a mirror layer has to be deposited on the lower surface of the MOIF (the one that is in contact with the sample). The reflected light then traverses back through the MOIF. A protective layer (generally some oxide or Si_3_N_4_ [32]) is also required to protect the soft mirror layer from scratching [19,31] (garnets themselves are rather hard and, thus, wear-resistant).

As mentioned above, the strengths of in-plane MOIFs are the absence of coercivity, which enables them to detect very low magnetic fields, and the absence of domains which would otherwise limit the spatial resolution [33]. In the absence of coercivity, the lowest detectable magnetic field is determined by the quality of the optical system and can be improved using image-processing or signal-processing techniques [15,34,35]. As a result, a field sensitivity as high as 10 μT can be achieved [36]. As there are no domains, the resolution limit is determined by the limiting magnetic inhomogeneity scale $\Delta \sim 2\pi \sqrt{A/K}$, where *A* is the exchange and *K* the anisotropy constant, which is ~0.1 μm for typical garnet constants [33]. Of course, because of the resolution limit of optical microscopes and the finite thickness of MOIFs, the spatial resolution in real experimental conditions is lower. The actual resolution also depends on the magnetic pattern being observed; its precise determination requires complicated calculations using a transfer function [14], but a rough estimate of the resolution in the range of the order of the MOIF thickness generally works well [33].

Finally, another advantage of in-plane MOIFs, which also owes its origin to the absence of domains, is their completely reproducible and reversible response to magnetic fields. This makes it possible to precisely calibrate them and, thus, they can be used in various quantitative measurements and sensor applications [14,15,33]. All this makes in-plane MOIFs a very good choice to be used as a standardized technique for local measurements of stray magnetic fields at the micrometer to millimeter scales. More details on in-plane MOIFs, including their fabrication, various applications, and calibration procedures, are given below.

## 4. Fabrication of Ferrite-Garnet MOIFs

High-quality single-crystalline ferrite garnet films are mainly prepared on gallium gadolinium garnet (GGG) substrates with the method of liquid-phase epitaxy (LPE), developed in the 1970s during the era of bubble domain technology [16,17,18]. As mentioned above, the magnetization direction in the films is determined by the minimum of magnetic free energy, which includes cubic and uniaxial anisotropy contributions, the interaction with the external magnetic field, and the energy in the self-demagnetizing field [13]. Cubic anisotropy can be adjusted and brought close to zero by doping with transition d-metal ions (Co^2+^, Ru^3+^, Ru^4+^, Os^3+^, Rh^4+^, and others). The uniaxial anisotropy amounts mainly to the magnetoelastic anisotropy, which depends on the lattice mismatch between the film and GGG substrate and also varies depending on the transitional metal ions [13,32]. The lattice mismatch can be controlled by the growth parameters such as supercooling [13]. As a result, both positive and negative values of the uniaxial anisotropy can be obtained, resulting in MOIFs with perpendicular or in-plane anisotropy, respectively. Thus, to prepare films with the desired anisotropy, one has to select the charge composition and adjust the supercooling. Still, often, it is difficult to achieve a high level of reproducibility, so one or two reference processes may be needed to fine-tune all the parameters [13]. As a result, one can prepare a garnet film with the desired properties.

## 5. Calibration

**Figure 2:**
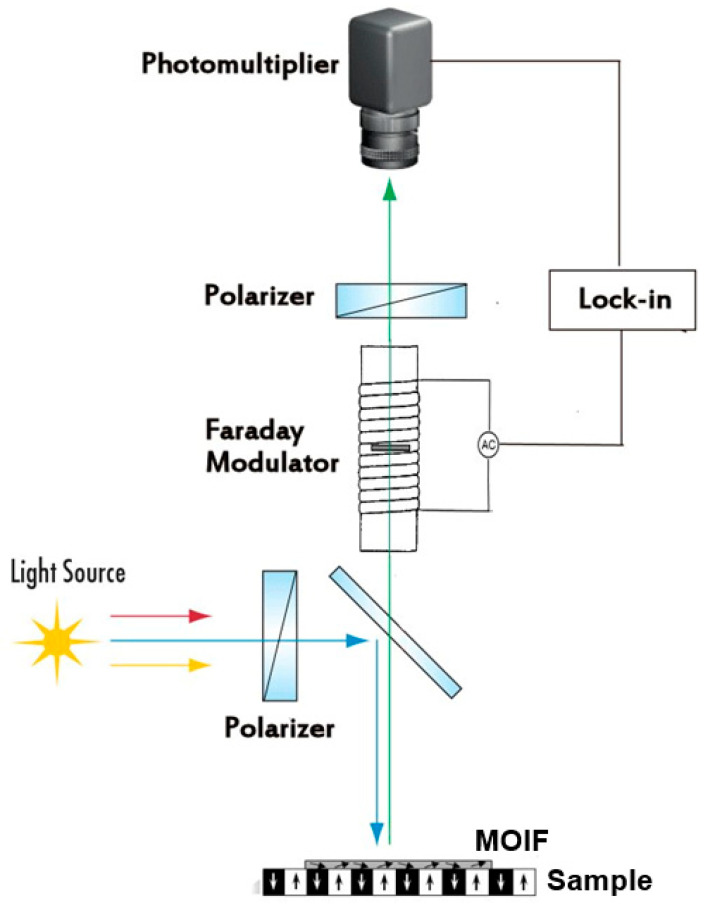
Schematic diagram of magneto-optical setup. The role of the Faraday modulator is explained in Section 5.3 below. The blue arrows indicate the incident polarized light and the green arrow indicates the light reflected back from the MOIF’s mirror layer. (The red and yellow arrows just indicate that the light is not monochromatic) [15].

The absence of coercivity and the reversible response of in-plane MOIFs make them a perfect candidate for use as quantitative sensors which can be calibrated and used for traceable measurements of the local magnetic field. These sensors, together with analyzing and imaging optics, transduce the measured field strength into a light intensity which can be measured using digital cameras or photomultiplier tubes (PMT). The fundamentals of the underlying magneto-optical effects are discussed in [37] and in a recent review by Sato et al. [38]. The analyzing optics, typically, are composed of two polarizers that polarize the incoming light beam and convert the rotation of the reflected light polarization into an intensity, respectively (Figure 2). The intensity of light passing through crossed polarizers in a polarizing microscope is described by the Malus law [13,39].

(3) $$I_{\pm }=\left(1-\xi \right)I_{0}\exp\left(-\alpha l\right)[sin^{2}\left(\beta \pm \delta_{F}l\right)+\xi ],$$

where β is the angle of the polarizer/analyzer uncrossing; $I_{0}$ is the incident light intensity; the factor exp(−αl)  characterizes the attenuation of the light in the MOIF material with an absorption coefficient α and path length l; $\delta_{F}l$ is the angle of Faraday rotation in the MOIF, and the extinction factor ξ arises from the imperfectness of the microscope’s optical system. Symbol ± stands for the two possible directions of Faraday rotation where it either adds up to or is subtracted from the effect of polarizers’ uncrossing. The main cause of the non-negligible extinction factor is the imperfectness of the polarizers. Normally, if prism polarizers are used, ξ is close to zero. However, at high magnifications, the polarized light image becomes deteriorated and it cannot be neglected.

### 5.1. Two-Dimensional Calibration Using Digital Cameras

Equation (3) can be inverted to find the Faraday rotation, $\delta_{F}l$, as a function of the light intensity which can be measured using digital CCD or CMOS cameras [40]. As the Faraday rotation varies, with good approximation, linearly with magnetic field, this allows one, in turn, to find the local magnetic field In practice, image processing is used to subtract the background first, which eliminates the effects of the various imperfections of the optical system. Then, the Malus law (3) can be applied, and its inverse form can be used to find the local magnetic field. As some of the constants in (3) are generally not known, they are used as fitting parameters to achieve the best match with the calibration curves (i.e., curves measured with the MOIF placed in a homogeneous field). Thus, the exact form of the functional relation between the measured light intensity and the magnetic field can be obtained. Then, it can be applied to derive stray field maps around magnetic samples. When this procedure was first developed back in the 1990s, the same values of the parameters were used for each pixel of the image [40]. This approach would leave various inhomogeneous effects unaccounted for, such as inhomogeneous illumination in the microscope, the inhomogeneities of the MOIF sensor, the imperfections of the optical system, etc. Lately, with the improvement in computational power, it has become possible to perform the same procedure pixel by pixel so that each pixel has its own calibration curve and, thus, all inhomogeneity effects are accounted for [14]. Alternatively, the correction of lighting artefacts is possible by analyzing data from images taken at different analyzer positions [41,42].

Equation (3) has a quadratic form which imposes some difficulties on both measurements and the calibration of the measurement setup for crossed polarizers, i.e., for β=0. Namely, around zero, a quadratic function has a zero derivative. That is, the sensitivity of the MO setup in low fields should be very low. Additionally, due to the symmetry of the quadratic function, positive and negative fields produce the same contrast and cannot be distinguished. A simple solution is to uncross the polarizers so that the angle *β* is nonzero. This shifts the working point away from the minimum so that the derivative of (3) is no longer zero and, thus, solves both issues mentioned above. The optimal angle of uncrossing, *β*, depends on the maximal Faraday rotation in the MOIF and is generally around a few degrees [13,19].

Recently, a polarization camera was presented that integrates an image sensor with a micropolarizer array, which allows for direct measurements of the angles of polarization rotation and which is, thus, insensitive to changes in the light intensity [43].

### 5.2. Differential Calibration Scheme

Equation (3) for the light intensity has a maximum derivative (resulting in the highest sensitivity of the MO setup) and has a linear form (thus making the calibration process easier) for the polarizers’ uncrossing angle $\beta =45^{{}^{\circ}}$. However, this results in the high intensity of the transmitted light which may reduce the dynamic range of some digital cameras if their depth of quantization is not high enough [13,33,39]. Additionally, this may result in larger intrinsic noise. These issues can be resolved by applying a differential scheme where the light beam is split into two after passing through the MOIF. The two split beams then pass through two independent second polarizers (analyzers) and are registered using two digital cameras. Then, the two images are digitally subtracted. If the two polarizers are uncrossed by equal but opposite angles ±*β*, the resulting differential signal will vary linearly with the Faraday rotation in the MOIF [13] (*β* does not have to be 45°; the response will be linear for any *β* but the sensitivity is higher for higher *β* values). The linear response makes the calibration procedure much simpler and solves the issue of low sensitivity around the minimum of the quadratic function. Moreover, the differential scheme eliminates the background with its inhomogeneities and reduces, to a large extent, the effect of inhomogeneous illumination [13].

Another approach, implemented in [44,45], is to use a Faraday rotator placed after the first polarizer. The Faraday rotator consists of a prism of doped glass or TGG (terbium gallium garnet) or some other material with a high Verdet constant placed inside a solenoid. By passing a current through the solenoid, one can induce a bias in the Faraday rotation and, thus, shift the working point away from the minimum in Equation (3). Another differential polarimeter switching the bias Faraday rotation by an angle +α and −α synchronously with the video frame rate was developed in [46].

### 5.3. Analog Calibration Using Faraday Modulator

An alternative approach developed in [33] makes use of a Faraday rotator installed after the polarizer, similar to that described in [44,45]; see Figure 2. However, instead of a DC field, a small AC field was generated in the rotator’s solenoid, resulting in the modulation of the total Faraday rotation (MOIF plus rotator). Thus, the term Faraday modulator was used. The resulting AC signal was then measured using a PMT connected to a lock-in amplifier. A small modulation applied to a permanent signal works effectively as an analog differentiation scheme. Thus, the quadratic form of Equation (3) was converted to a linear form. As discussed above, the linear form is devoid of all drawbacks of the quadratic signal, such as low sensitivity around the minimum, the impossibility to distinguish positive and negative fields of the same magnitude, and a complicated procedure for the evaluation of calibration curves. Moreover, it was shown in [33] that the second harmonic measured by the same lock-in amplifier is independent of the Faraday rotation and, thus, can be used as a reference signal to eliminate interferences such as imperfections in the MOIF or light instability.

The obvious advantage of this calibration scheme is its simplicity. Magnetic field values can be derived from the signal in real time without any complicated computations. Another important advantage is the high signal-to-noise ratio due to the use of a lock-in amplifier, which allows one to achieve a high sensitivity and resolution. A sensitivity of 10 μT was demonstrated in [36]. At the same time, this approach incurs the disadvantage that PMT measurements can be performed only at a single spot; thus, time-consuming scanning must be performed to obtain 2D field maps. Although, at the same time, scanning measurements present another advantage that, as all measurements are performed at the same spot in the field of view there is no effect of illumination inhomogeneity [33].

Given all the weak and strong attributes of the different measurement techniques described above, the choice of the best technique depends on the specific application. Where the speed of measurements is important, digital cameras should be used. However, to achieve a high sensitivity and resolution with respect to the magnetic field, the analog modulation technique may become preferable.

### 5.4. Effect of In-Plane Field Component, In-Plane Domains, and Sensor Thickness

Mostly, the calibration curve of MOIFs is obtained using measurements in a uniform field normal to the indicator surface. However, strictly speaking, this approach is incorrect as the stray fields of inhomogeneously magnetized samples include both normal and parallel components. This was first pointed out in [47], where the effect of the in-plane field component was demonstrated by applying in-plane fields with different magnitudes in addition to a uniform normal field. The authors developed an inversion scheme that takes into account the effect of both normal and in-plane field components and, thus, were able to obtain more realistic current distribution profiles in superconductors compared to those calculated when neglecting the effect of the in-plane component. In recent works, the effect of the in-plane field component was studied in more detail [14,15]. It was shown that in lower fields, approximately up to a half of the MOIF’s saturation field, neglecting the in-plane component is justified as it does not have much effect on the MOIF response, while at higher fields, the in-plane component must be taken into consideration. At higher fields, where the in-plane field component cannot be neglected, the procedure to separate the field components becomes quite complicated because there are two unknowns (the in-plane, $H_{∥}$, and normal, $H_{⊥}$, field components), while only one quantity (light intensity) is measured at each pixel. One solution was proposed in [15], where a small bias in-plane field was applied and the respective change in the light intensity caused by this bias field was measured. This change in the light intensity constitutes the second measured quantity and, thus, both unknowns,$\;H_{∥}$ and $H_{⊥}$, can be found. The procedure is, however, rather complicated because the resulting equations cannot be solved analytically and require a numerical solution for each pixel. An alternative forward simulation approach, based on a preassumed distribution of the sample stray field used to calculate the MOIF response via a transfer function, was developed in [14]. This approach also involves complicated computations for each pixel, so there is no easy way to take the in-plane field component into account. Thus, the easiest way out is, wherever possible, to use MOIFs with higher saturation fields so that the stray fields produced by the sample should be below half the saturation field. As mentioned above, in that case, the in-plane field component can be neglected, and the normal component can be directly derived from the measured light intensity. Note that even in cases where the in-plane component is negligible for the MOIF measurements, the full magnetic field vector can be calculated from the normal magnetization component using a transfer function in Fourier space [43].

Another factor, apart from the in-plane field component, that limits the validity of MOIF calibration in a uniform field is the finite thickness of MOIFs. As the stray fields above inhomogeneously magnetized samples decay strongly with the distance from the sample surface, the MOIF produces an integrated response that represents a kind of average field over the MOIF’s thickness. This effect becomes increasingly relevant for larger ratios of MOIF thickness to magnetic structure size. The thinner the MOIF, the more precisely one can say what height the measured field corresponds to. However, reducing the MOIF thickness decreases the sensitivity and worsens the signal-to-noise ratio; thus, using thinner sensors is not always justified. To take the sensor thickness into account, a transfer function approach [14] can be used. Thus, both the in-plane field component and the finite sensor thickness can be accounted for, and a complete calibration of the MO sensor can be performed for real conditions of inhomogeneous stray fields.

**Figure 3:**
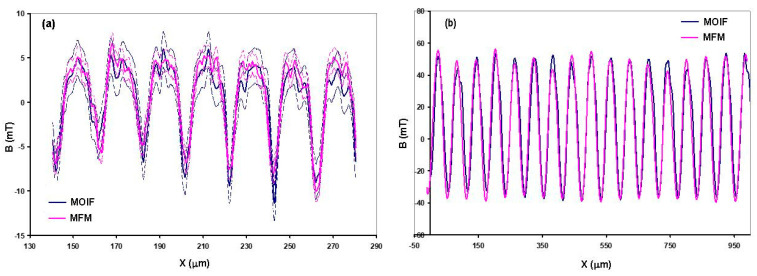
(**a**) MFM−MOIF comparison on thermomagnetically patterned NdFeB sample with 20 μm periodicity. (**b**) MFM−MOIF comparison measurements on SENSITEC GmbH lithographically patterned NbFeB reference sample with 60 μm periodicity performed using TUBITAK’s high-resolution magneto-optical system and PTB’s large range MFM. Both datasets show good agreement, thereby validating the calibrations of the systems. Reproduced with permission from [36] Hans Werner Schumacher, 2019.

Both approaches described above (applying a bias in-plane field [15] or the forward simulation approach using a transfer function [14,43]), although complicated, now allow one to perform traceably calibrated measurements of spatially inhomogeneous fields using MOIF sensors. These traceable measurements can be validated by comparing with another traceable spatially resolved technique. Such comparisons performed on industrial magnetic scales using MOIF and quantitative magnetic force microscopy (qMFM) have demonstrated excellent agreement (Figure 3) [14,36]. That is, due to the latest theoretical advancements, MOIFs can now be used for validated traceable measurements of local magnetic fields for industrial artifacts and in scientific research.

It should also be mentioned that in-plane MOIFs are not completely free of domains. In fact, there are very large, several mm wide domains with the magnetization vector having different orientations within the film plane. Normally, those domains are barely visible; in any case, being large, they do not impact the out-of-plane magnetization rotation and do not interfere with the images observed at microscopic scales [27]. However, in the areas of strong field gradient, they become smaller and their MO contrast becomes stronger [13,33]. Thus, one should take care when visualizing strongly inhomogeneous field distributions where the in-plane domains may interfere with the image.

In conclusion to this section, one should also notice that MOIF parameters are temperature-dependent [14,36]. Although this dependence is not strong and can be ignored for most room-temperature applications, there are some industrial and scientific applications that require high temperatures up to about 150 °C or cryogenic temperatures. In such cases, MOIF calibrations have to be performed at the same temperature as where the MOIF measurements will be carried out. This is essential for both homogeneous field calibrations and the forward simulation approach [14], where all MOIF parameters used for the simulation, such as anisotropy constants, the Verdet constant, and saturation magnetization, have to be determined for the specific temperature.

## 6. Applications of MOIF Technique

The first magneto-optical imaging films were yttrium iron garnet films developed for magnetic memory applications back in the 1970s [16,17,18], although those developments never reached the stage of practical applications. The first in-plane MOIFs were developed about a decade later for recovering damaged magnetic recordings in the black boxes of crashed planes. Just at that time, high-temperature superconductors were discovered, which have very rich magnetic properties. That boosted the interest in magnetic imaging techniques and especially in the MOIF technique due to its simplicity and high sensitivity [20,21,22,23,24,25,26]. Due to their high resolution and the ability to perform traceable quantitative measurements, MOIFs were used to reconstruct the distribution of critical currents in superconductors; see, e.g., [33,40,48]. The MOIF resolution appeared to be even sufficient to distinguish individual Abrikosov vortices in superconductors [44,49,50]. Moreover, apart from observations, the manipulation of single vortices by magnetic domain walls was demonstrated [49].

Due to their application in high-temperature superconductors, MOIFs became widely known to the scientific and industrial communities. This fostered their use in various areas of research and industrial applications. Thus, eddy current imaging using MOIFs was developed for nondestructive testing [51,52,53,54]. Similarly, MOIFs were used for current imaging in integrated circuits as a quick diagnostics tool that allows one to visualize leakage currents [55]. Surface current densities as low as 0.1 mA/μm were reported to be observed using magneto-optics. MOIFs with a tailored Curie temperature were used for thermomagnetic imaging [56] which also allowed one to perform the quick and precise diagnostics of integrated circuits.

Apart from diagnostics and nondestructive testing, there is a wide range of well-established and prospective applications of MOIFs. Some examples include the evaluation of hybrid magnetic shape memory microactuators [57], the manipulation of functionalized microbeads [58], and forensic applications (hidden magnetic images in documents and banknotes) [13]. MOIFs can be used for the traceable characterization of the field distribution of magnetic scales [14,15], which can, thus, be validated for usage in magnetic encoders for high-precision magnetic positioning. There are a lot of scientific applications which include, of course, the observation of domain structures in magnetic materials [27,59,60,61], the observation of domain wall movement under stress [62], stray fields induced by single domain walls in soft magnetic materials [63], and studies of magnetic-phase transitions [64,65], the magnetic structure of granular films and multilayers [66], and superconductor/ferromagnet hybrid structures [67]. Calibrated external bias fields can be applied in situ to expand the measurement range and to minimize the measurement errors [68]. MOIFs are successfully used to study magnetization processes in magnetic functional devices such as spin valves [69], magnetic tunnel junctions [70], and lab-on-chip micromanipulators [71], giving valuable information for the further development of such devices. Due to the very narrow linewidth of ferromagnetic resonance, MOIFs can also be used in microwave applications [72].

By using a Bi2:NIG (neodymium iron garnet) MOIF film and green and yellow LED, a color contrast for the magnetic field with color scales indicating the quantitative values of magnetic fields could be obtained [73]. Large MO imaging plates with an area of 11 × 15 cm^2^ were prepared from Bi2.5:NIG on a glass substrate for large-scale imaging [74].

Due to the flat shape of MOIFs, they are normally used for the visualization of stray fields above objects with a flat surface; however, it was shown in [75] that magnetic domains can also be observed in nonflat objects such as magnetic wires. Additionally, amorphous MOIFs can be grown on different kinds of substrates, including flexible ones, to improve contact with the sample or, in some cases, directly on the studied sample [13]. Apart from microscopy observations, large-area MOIF observations can be performed using low-magnification optical setups and scanning techniques [31,76].

In the abovementioned applications, both industrial and research, mostly in-plane MOIFs were used due to their advantages described in this review article. However, as already mentioned, perpendicular MOIFs with a domain structure can operate as optical contrast “amplifiers” and, thus, can be used to visualize magnetic fields with mostly in-plane and only weak perpendicular components, which would produce a very weak contrast using in-plane MOIFs. Thus, perpendicular MOIFs were used to observe the domain structures and real-time remagnetisation processes in grain-oriented electrical steel sheets [27,77,78,79]. Moreover, MOIFs appear to be a good tool to study the effect of mechanical stress on the domain structure as the MOIF placed on top of the sample remains unaffected by the stress [77,78]. Perpendicular MOIFs were also used for studies of micropatterned hard magnetic films for MEMS applications [80].

Due to the ability to quantitatively image the stray field distribution over square-centimeter-sized areas with subsecond temporal resolution, MOIFs have great potential to become a standard tool for quality control in industrial materials, such as steel sheets and magnetic scales, and to complement the existing international IEC standard for nanoscale magnetic field measurements, which is presently based on magnetic force microscopy (MFM) [81].

## 7. Conclusions

Magneto-optical indicator film (MOIF) sensors represent an easy tool for observation and quantitative measurements of stray magnetic fields with a resolution limit down to a fraction of a micrometer and a size of observation area of up to several centimeters; thus, this technique is capable of bridging macroscopic magnetic field measurements with microscopic measurements such as MFM. MOIFs can be easily calibrated and made traceable to primary standards of magnetic field. At the same time, the ease of operation makes them an instrumental tool for many industrial applications, such as nondestructive control, product development, the optimization of magnetic materials, etc., as well as for research applications. The underlying physics of MOIF functioning have been fully described only recently and, accordingly, verifiable calibration approaches have been developed and their validity for industrial applications has been demonstrated. The present review encompasses all these achievements, thus presenting a complete picture for potential applications in both industry and research.

## Data Availability

No new data were created or analyzed in this study. Data sharing is not applicable to this article.
